# MXenes and Their Applications in Wearable Sensors

**DOI:** 10.3389/fchem.2020.00297

**Published:** 2020-04-21

**Authors:** Ming Xin, Jiean Li, Zhong Ma, Lijia Pan, Yi Shi

**Affiliations:** Collaborative Innovation Center of Advanced Microstructures, School of Electronic Science and Engineering, Nanjing University, Nanjing, China

**Keywords:** MXenes, strain sensor, pressure sensor, biosensor, gas sensor

## Abstract

MXenes, a kind of two-dimensional material of early transition metal carbides and carbonitrides, have emerged as a unique class of layered-structured metallic materials with attractive features, as good conductivity comparable to metals, enhanced ionic conductivity, hydrophilic property derived from their hydroxyl or oxygen-terminated surfaces, and mechanical flexibility. With tunable etching methods, the morphology of MXenes can be effectively controlled to form nanoparticles, single layer, or multi-layer nanosheets, which exhibit large specific surface areas and is favorable for enhancing the sensing performance of MXenes based sensors. Moreover, MXenes are available to form composites with other materials facilely. With structure design, MXenes or its composite show enhanced mechanical flexibility and stretchability, which enabled its wide application in the fields of wearable sensors, energy storage, and electromagnetic shielding. In this review, recent progress in MXenes is summarized, focusing on its application in wearable sensors including pressure/strain sensing, biochemical sensing, temperature, and gas sensing. Furthermore, the main challenges and future research are also discussed.

## Introduction

Wearable devices possessing excellent mechanical compliance and unprecedented sensitivity are attracting vast interest as the next-generation interactive platform for health monitoring, motion detection, robotics, and prosthetics (Khan et al., 2016; Heikenfeld et al., 2018; Bandodkar et al., 2019; Li N. et al., 2019; Yang J. C. et al., 2019). Flexible electronics are in need of surface-mounted wearable devices to fit the complex structure of objects with reliable electrical characteristics under cyclic strain conditions during daily movements (Ray et al., 2019), which is beyond the capability of conventional silicon-based rigid electronics. To this end, researchers have proposed strategies from external circuit structural design, e.g., serpentine mesh metal traces (Xu et al., 2014), to the internal microstructure of device design, e.g., mechanical sensing of pyramid microstructure (Mannsfeld et al., 2010). Although these techniques showed their feasibility in different applications, various challenges still exist in (1) the trade-off between mechanical flexibility and electrical performances (i.e., most materials achieve greater flexibility with its degradation in carrier mobility). (2) The lack of a scalable fabrication process. (3) The presence of local structural surface defects of nanomaterials like carbon nanotubes. Therefore, new materials are anticipated to be discovered for the further development of wearable applications.

**Figure 1:**
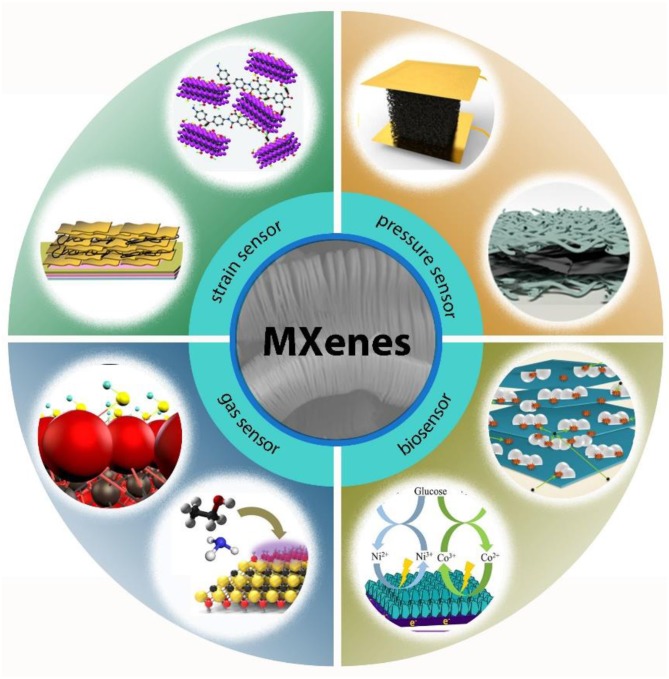
An overview of the applications of MXenes in the wearable devices field. Reproduced from Cai et al. (2018) with permission from American Chemical Society; reproduced from Liu et al. (2018) with permission from WILEY-VCH Verlag GmbH & Co; reproduced from Li X.-P. et al. (2019) with permission from Elsevier Inc; reproduced from An et al. (2018); reproduced from Wang et al. (2019); reproduced from Li M. et al. (2019) with permission from Elsevier B.V; reproduced from Kim et al. (2018) with permission from American Chemical Society; reproduced from Lee et al. (2017) with permission from American Chemical Society.

In 2011, the birth of MXenes introduced a new family into the two-dimensional (2D) materials and was further proved to be promising in the wearable sensory applications due to its controllable preparation method and fascinating properties. In essence, MXenes are a group of 2D early transition metal carbides, nitride, or carbonitrides prepared by selectively etching of the group IIIA or IVA element from the three-dimensional (3D) MAX phases. The 3D MAX phases are indicated due to the composition: M_n+1_AX_n_ layers (n equals to 1, 2, or 3), in which “M” stands for early transition metal (including Ti, Nb, Cr, Mo, etc.) and “X” is carbon and/or nitrogen that is connected with layers of A atoms, named from the main group element (group IIIA or IVA) (Ma and Sasaki, 2010; Naguib et al., 2011, 2013; Khazaei et al., 2013). As a new star of 2D materials, MXenes combine the metallic conductivity of transition metal carbides/carbonitrides with the hydrophilic nature of their terminated surfaces which is uncommon. Their preparation process makes the surface of MXenes rich in functional groups, and their unique accordion-like appearance endows MXenes with attractive electronic, optical, and magnetic properties, which can draw inspiration in energy storage (Lukatskaya et al., 2013; Ghidiu et al., 2014; Anasori et al., 2017), electromagnetic shielding (Shahzad et al., 2016), and sensing (Chen et al., 2015). As shown in Figure 1, with the inspiration of MXenes's fascinating electrical and biological characteristics, researchers began to combine MXenes with wearable devices to obtain sensors with different capabilities.

Herein, we especially focus on MXenes's characteristics as flexible electronics and provide insights into the relevant study. Beginning with summarizing MXenes's synthesis, modifications, and properties, this article reviews several potential applications in wearable sensors of force perception, biomedical analysis, and gas sensors. We also provide discussion about current challenges and outlook into future development.

## Synthesis and Electronic Properties

MXenes are prepared by selective etching of the A element layers from the 3D MAX phases which is a large group of the ternary carbides and nitrides. The preparation and modification of MXenes are of great concern, especially in the field of sensors. The synthesis can effectively control the morphology and surface termination of MXenes, which have a great effect on sensory functions. For example, electrical properties are changed under mechanical force with the help of micro/nano morphology; multi-layer morphology is helpful to carry enzymes for biosensors and allow fast diffusion of targeted molecules. The preparation methods of MXenes are various [e.g., bottom–up synthesis methods (Shahzad et al., 2016), synthesize MXenes from non-MAX-phase precursors (Meshkian et al., 2015), etc.] and MXenes possess multifrequency properties (e.g., optical, and magnetic properties), in this section, from the point of view of sensors, we focus on the wet etching (etching with hydrofluoric acid) and electrical properties of MXenes.

### Synthesis

**Figure 2:**
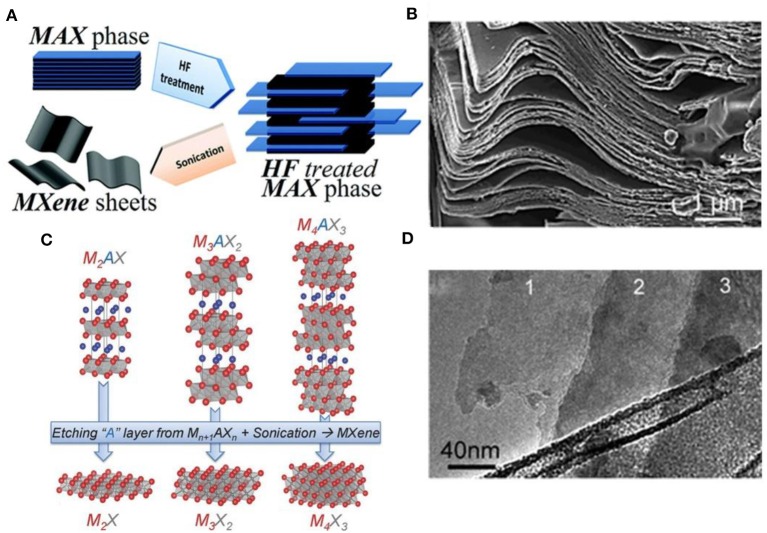
**(A)** Schematic for the exfoliation process of MAX phases and formation of MXenes; **(B)** The image of the multi-layer of MXenes; **(C)** Structure of MAX phases and the corresponding MXenes; reproduced from Naguib et al. (2013) with permission from WILEY-VCH Verlag GmbH & Co. **(D)** The image of the single-layer of MXenes. **(A,B,D)** are reproduced from Naguib et al. (2012) with permission from American Chemical Society.

Thanks to the relatively weaker strengths of the M-A bonds than M-X bonds, it is possible to remove the more chemical-active “A” atoms without destroying M_n+1_X_n_ layered structures. Naguib and colleagues first used aqueous hydrofluoric acid (HF) to replace Al atoms by surface terminations, including hydroxyl (-OH), oxygen terminated surfaces (-O), or fluorine terminated (-F), from Ti_3_AlC_2_ and separated graphene-like single sheet M_n+1_X_n_ layers which labeled MXenes. They provided a typical process to prepare MXenes: the precursors were firstly treated with etching solution (HF or acid-fluorides), the specific concentration mixture is centrifuged or filtered until the solid-liquid separation, washed with deionized water until the pH value of the supernatant solution is between 4 and 6 and were subsequently treated with shearing forces or sonication to obtain a single-layer stack (Naguib et al., 2014; Alhabeb et al., 2017). With this process (Figure 2A), the morphology of MXenes can be controlled by adjusting the concentration of etching solution, etching time, ultrasonic time (Malaki et al., 2016), and experimental temperature (Naguib et al., 2012; Persson et al., 2018). According to the principle, more than 20 different compositions of single-layer MXenes or multi-layer stacks (Figures 2B,D) have been experimentally obtained by this process. Although recently, different synthesis strategies (Rasid et al., 2017) have been developed including chemical vapor deposition (CVD) (Gogotsi, 2015; Xu et al., 2015; Wang et al., 2017), template method (Jia et al., 2017; Xiao et al., 2017) and plasma-enhanced pulsed laser deposition (PEPLD) (Zhang et al., 2012), wet selective etching is still the main method of MXenes-based sensor fabrication.

The etching is a dynamic control process, and each kind of MXenes needs different etching time to achieve complete conversion. In general, MXenes with larger n in M_n+1_C_n_T_x_ need stronger etching and longer etching time. For example, under the same etching conditions, the etching time required for Mo_2_Ti_2_AlC_3_ (*n* = 3) is twice that of its *n* = 2 counterpart (i.e., Mo_2_TiAlC_2_) (Anasori et al., 2015). Each new kind of MXenes can be made under different etching conditions, resulting in different quality (defect concentration and surface termination). At present, since HF is still the mainstream etching solution, the characteristics of corrosiveness to the organism, dangerous treatment, and recovery hinder the batch synthesis and application of MXenes. Several synthesis strategies have been explored to avoid or minimize the utilization of HF. The most widely used is the mixture of hydrochloric acid (HCl) and fluoride salt, which forms HF in *situ* (Ghidiu et al., 2014). Other fluoride salts have also been used successfully (NaF, KF, and NH_4_F) (Liu et al., 2017a,b), along with different HCl concentration and lithium fluoride (LIF)/HCl molar ratio (Alhabeb et al., 2017). The fluorine-free MXenes can be obtained by hydrothermal treatment of Ti_3_AlC_2_ powder in the alkali solution (Li et al., 2018). The electrochemical etching of Ti_2_AlC in diluted HCl can yield MXenes without F terminations (Sun et al., 2017). These synthesis strategies pave the way for the preparation of biocompatible wearable sensors.

The appearance of MXenes etched by the wet method is the accordion-like shape. Single-layer or few-layer MXenes can be obtained by ultrasonic treatment, which possess a high aspect ratio. Another method is liquid exfoliation by the intercalation of molecules to obtain colloids with high yield (Mashtalir et al., 2013; Ghidiu et al., 2016). The introduction of appropriate molecules can cause the expansion of the interlayer space, and weaken the interaction between layers, which can split the multilayer into a single sheet. Both methods utilized depend on the etching method and MXenes composition (Verger, 2019). In all cases, the single MXenes layer is less than 1 nm thick (3,5,7 atomic layers) (Figure 2C), with up to tens of microns in the lateral dimension of microns (Huang et al., 2018).

### Electronic Properties

The electronic properties of MXenes are the most unique properties compared to other 2D materials, such as graphene. Recent research demonstrated that the metal layers (M-layer) are the main factor to affect the electronic properties of MXenes (metallic conductivity of Ti_3_ C _2_ compared with Mo_2_ TiC _2_) (Lipatov et al., 2016). It is confirmed that a delicate balance between temperature and the activity of the etchant needs to be maintained and the electronic behavior from metallic to semiconductor-like can be regulated by changing the two outer transition M-layer of a 2D carbide (Anasori et al., 2016). The electroconductibility can also be influenced by the fabrication methods, because of the surface termination and different extent of defects. The more defects exist, the low electroconductibility MXenes possess, which is due to the destruction of the ordered structure for free movement of electrons. Defects can be controlled by doping different atoms. Through the exchange of ions with different electric densities, the defects of charge imbalance will be produced (Feng et al., 2017). Because most of the etching solution is fluoride, the surface of MXenes usually contains -OH, -O, and -F functional groups. With the surface termination changed (e.g., small molecule adsorption), the electrical conductivity of MXenes has changed accordingly (Zha et al., 2015).

As the early transition metal carbides and carbonitrides, MXenes not only have the conductivity comparable to copper but also have the properties of carbon or nitride. Energy band structure and electronic density of state (DOS) of MXenes have been studied extensively by density functional theory (DFT). The bare MXenes single-layer is predicted to be a metal layer with high electron density close to the Fermi level (Tang et al., 2012; Khazaei et al., 2013). With the sensitive electrical properties, MXenes can be used to detect strain through the crack mechanism, When the MXenes-based sensor is cracked by strain, its conductivity/resistivity will change with the increase of crack. MXenes can be applied in biosensors to detect small molecule adsorption and sensitive detection of several gases. With different composition and concentration, molecules adhere to the MXenes surface termination, the conductivity will change accordingly (Yu et al., 2015). Different molecules have selective adsorption by MXenes materials with different compositions, and their conductivity changes obviously.

Appropriate surface modification/functionalization can enhance its sensing performance and biomedical properties. Through physical absorption or electrostatic attraction, molecules, or atoms with different structures can be selectively adsorbed on the surface of MXenes, which will affect their own electrical properties to achieve the purpose of sense. For instance, the biocompatible polymers, such as soybean phospholipid, are more suitable for surface modification of MXenes because of their large surface area and biodegradability (Dai et al., 2017a,b). MXenes usually display a negative charge because their surface terminals are rich in -OH, -O, and –F functional groups (Khazaei et al., 2013; Shein and Ivanovskii, 2013; Berdiyorov, 2015). The molecules with a positive charge are adsorbed on the surface of MXenes by electrostatic force, enabling the composite with enhanced sensing capabilities (Li S. et al., 2019) or drug transport (Liu G. et al., 2017).

## Physical Sensor

MXenes prepared by chemical liquid etching usually have various functional groups on their surface with strong hydrophilicity and ease in chemical modification. Meanwhile, Mxenes have many excellent properties, such as electronic properties and bending strength comparable to graphene, and the oxidation resistance and electron irradiation resistance superior to graphene (Enyashin and Ivanovskii, 2012; Khazaei et al., 2013; Anasori et al., 2015). MXenes materials can be utilized in the stress sensor to detect tiny shape variables due to their excellent electronic properties. The structure of the accordion-like shape can be used in a super-*sensitive* piezoresistive sensor. MXenes materials can be easily mixed with other materials to improve sensor performance. Therefore, MXenes have gradually attracted more attention in the field of physical sensors.

### Strain Sensor

The flexible strain sensor transforms the tensile strain of the device into the resistance signal output (Ma et al., 2019). When the external force is applied to the sensor, the internal conductive materials or networks will crack due to external forces, which cause the electrical characteristics to be changed accordingly. The conductive network is usually made up of 2D sheets that are closely stacked. Interaction forces such as Vander Waals forces may exist between adjacent sheets. Therefore, under the action of external stress, the sheets cannot achieve effective sliding, but can only disperse the stress by producing large cracks. The crack dimension is proportional to the stress when the external force is strong enough, the conductive path will be blocked and greatly limits the improvement of sensing range and stability.

**Figure 3:**
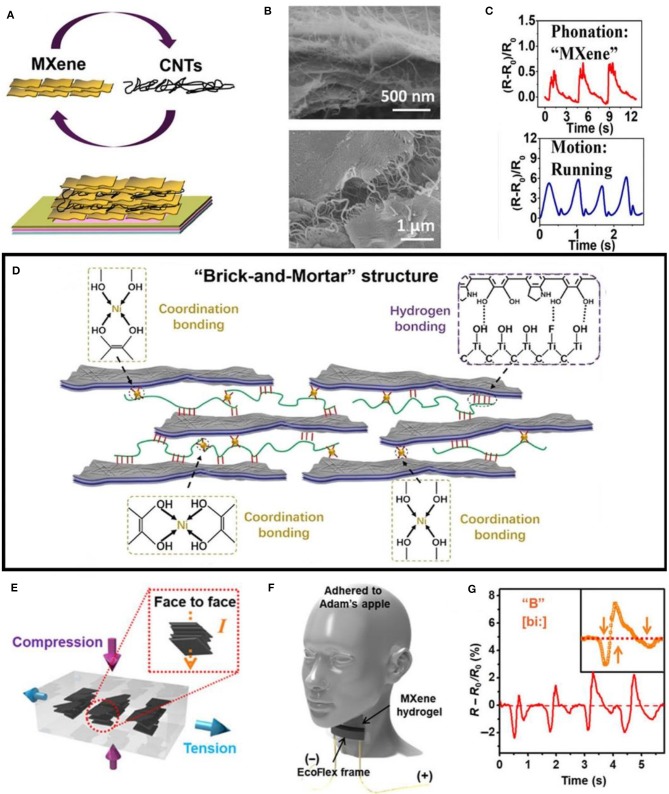
MXenes for strain sensor. **(A)** Fabrication process of a sandwich-like Ti_3_C_2_T_x_ MXene/CNT layer; **(B)** SEM images of sandwich-like Ti_3_C_2_T_x_ MXene/CNT layers. **(C)** Relative resistance responses of the sensor in phonation and motion; **(A–C)** are reproduced from Cai et al. (2018) with permission from American Chemical Society. **(D)** Schematic illustration of the Ti_3_C_2_T_x_-AgNW-PDA/Ni^2+^ sensor based on the “brick-and-mortar” architecture; reproduced from Shi et al. (2019) with permission from American Chemical Society. **(E)** Schematic illustration of the mechanism of the electromechanical responses of M-hydrogel; **(F)** Schematic for vocal sensing and **(G)** Resistance change in response to similarly sounding letters “B.” **(E–G)** are reproduced from Zhang et al. (2018).

To reduce the interaction between 2D materials and construct a new conductive network, it is a common method to add the second phase or the nth phase materials of different dimensions into 2D materials. For instance, Cai et al. (2018) utilized Ti_3_C_2_T_x_ MXenes flakes combined with hydrophilic single-walled carbon nanotubes (SWNTS) to fabricate sandwich-like sensing layers through a layer-by-layer (LbL) spray coating technique. The layers of Ti_3_C_2_T_x_ are in an orderly stacking state, and CNTs with high aspect ratio are disorderly distributed among the layers like fluff. The layers are woven together to form a complete conductive network (Figures 3A–C). The sensitivity (GF = (Δ R/R0)/ε) of the flexible strain sensor can reach 64.6 in the range of 0–30% strain and 772.60 in the range of 40–70%. By adding fluffy CNTs, the Ti_3_C_2_T_x_ MXenes flakes can be connected in a wide range of strain, which makes the sensor has a wide sensing range. Shi et al. drew inspiration from bionics, they combined silver nanowire (AgNW) with Ti_3_C_2_T_x_ and introduced dopamine (DPA) and nickel ions (Ni^2+^) to construct a nacre-mimetic strain sensor (Shi et al., 2019). Ti_3_C_2_T_x_ sheets and AgNW as “bricks” endow the whole composite system with high conductivity and mechanical brittleness, while PDA/Ni^2+^ as “mortal” connects “bricks” through various interface interactions (Figure 3D). The GF of this flexible strain sensor is 256.1, 433.3, 1160.8, 2209.1, and 8767.4 in the strain range of 0–15, 15–35, 35–60, 60–77, and 77–83%, respectively. The maximum sensing range is more than 50% and the sensitivity is higher than 200 in the whole range, which exceeds most of the reported flexible strain sensors. Thus, one-dimensional materials like a bridge, connect the MXenes sheets, which endows the device with high sensitivity and a wide strain sensing range.

Since pure Mxenes, like other 2D materials, in general, are not stretchable, adding polymer into MXenes can enhance its mechanical robustness as well as its sensing range (Ling et al., 2014). An et al. utilized Ti_3_C_2_T_x_ nanosheets, derived from the parent Ti_3_C_2_T_x_ MAX phase through MXenes and poly (diallyldimethylammonium chloride) (PDAC) to form composite films as LbL assembly (An et al., 2018). The conductivity of the film can reach 2,000 S/m, and it can be evenly loaded on various substrates like silicon, polydimethylsiloxane (PDMS), polyethylene terephthalate (PET), indium tin oxide (ITO), and glass. The strain sensor based on MXenes/PDAC composite membrane on PDMS can be stretched to 40%, while the bending sensor on PET can be bent 35%.

In addition to being integrated with other phase materials, it is also an effective method to build new microstructures by adjusting the morphology of MXenes. Yang Y. et al. (2019) utilized the common chemical liquid phase etching method to prepare Ti_3_C_2_T_x_ materials. By adjusting the etchant [HF and tetramethylammonium hydroxide (TMAOH)], etching time (6, 18, and 24 h), and ultrasonic time (20 min, 1~4 h), the morphology of Ti_3_C_2_T_x_ was effectively controlled, and the mixed network structure of Ti_3_C_2_T_x_ nanoparticles and nanosheets with different proportions was prepared to maximize the synergistic effect. The GF of the sensor is up to 178.4, 505.1, and 1176.7 in the strain range of 0–5, 5–35, and 35–53%, respectively and its maximum sensing range is 53%, which is suitable for all activities of the whole body.

In addition to the above materials used as flexible substrates, hydrogels are often applied in strain sensors because of their excellent stretchability and self-repair ability. In general, conductive materials enter the hydrogel to form conductive hydrogels. Zhang et al. (2018) utilized Ti_3_C_2_T_x_ and polyvinyl alcohol (PVA) hydrogel to form conductive MXenes-based hydrogels (M-hydrogel). Because of the cross-linking between the surface end groups of Ti_3_C_2_T_x_ and PVA hydrogels (Figures 3E–G), the hydrogel has a tensile strength of 3,400% and has a good self-repairing ability. The GF of the sensor in the range of 0–0.5 and 0.5–3.0% is 60–80 and 21, respectively. Different from the crack propagation mechanism, the sensor mainly changes the contact resistance between the Ti_3_C_2_T_x_ lamellae caused by the deformation of the hydrogel in response to the external force, to change the mechanical to the electrical signal. Liao et al. combined the prepared Ti_3_C_2_T_x_ sheets with hydrogels composed of polyacrylamide and polyvinyl alcohol to obtain MXenes nanocomposite hydrogel (Liao et al., 2019). Then, the composite hydrogel was immersed in the ethylene glycol solution to remove some water molecules. The MXene nanocomposite organohydrogel (MNOH) for strain sensing with high-sensitivity (GF = 44.85), antifreeze, and self-healing was developed.

### Pressure Sensor

The multi-layer MXenes with accordion-like shape and single-layer MXenes have been used for flexible piezoresistive sensors. When the pressure acts on the device, the pressure signal is converted into resistance signal output through the deformation of the material.

**Figure 4:**
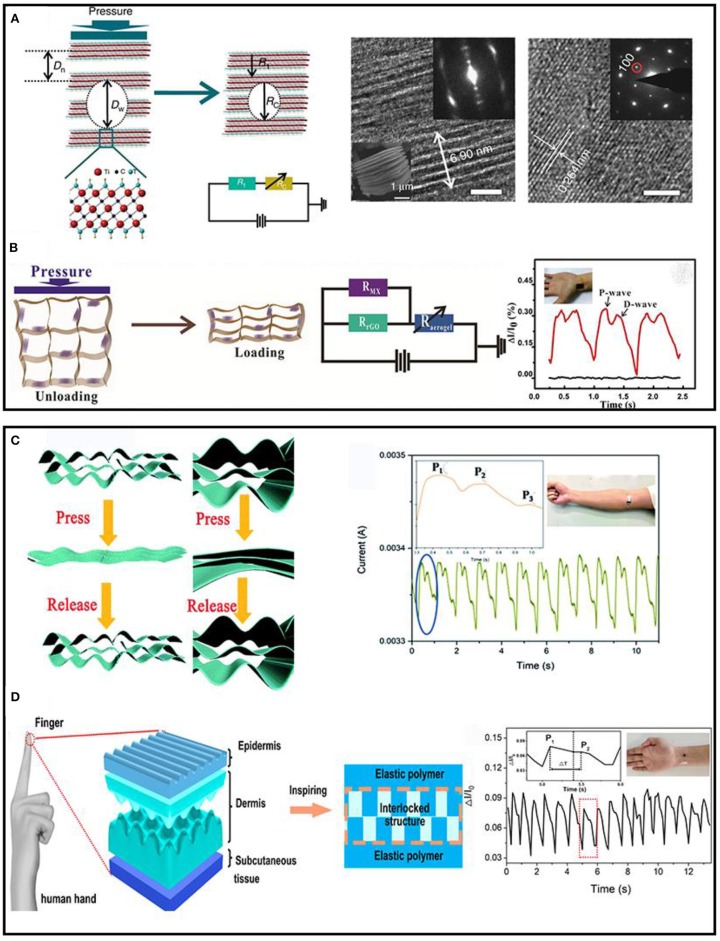
MXenes for pressure sensor. **(A)** Working micromechanism and SEM image of MXenes-material for piezoresistive sensor; reproduced from Ma et al. (2017). **(B)** Schematic illustration and application of MX/rGO aerogel sensor; reproduced from Ma Y. et al. (2018) with permission from American Chemical Society. **(C)** Schematic elasticity mechanisms and application of C-MX/CNC; reproduced from Zhuo et al. (2019) with permission from The Royal Society of Chemistry. **(D)** Schematic of interlocking structure of simulated human skin and the application of pulse measurement; reproduced from Wang et al. (2019) with permission from American Chemical Society.

In a multi-layer MXenes-based piezoresistive sensor, after the A-layer is removed by etching the MAX phase block, the accordion-shaped MXenes block is obtained. Each block is composed of several MXenes monolithic layers. Ma et al. (2017) fabricated a flexible piezoelectric sensor by coating the ethanol dispersed droplets of multilayer Ti_3_C_2_T_x_ on the polyimide (PI) integrated electrode. They first used the basic characteristics of greatly changed interlayer distances of MXenes under an external pressure for a real application (Figure 4A). The GF of the sensor is 180.1–94.8 and 94.8–45.9 in the range of 0.19–0.82 and 0.82–2.13%, respectively. Moreover, it can be used to explore the full-range human activities (e.g., eye blinking, cheek bulging, and throat swallowing). Because MXenes itself is very fragile and hard to sustain large pressure, MXenes need to be combined with materials with high mechanical strength as the skeleton to support the repeated stress and rebound of the sensor. There are mainly two kinds of MXenes-based flexible piezoresistive sensors, aerogel sensors, and MXenes/elastic matrix sensors. Aerogels have the characteristics of high porosity, ultralight, and superelasticity, making them excellent choices for fabricating flexible piezoresistive sensors. MXenes lamellae are usually unable to form aerogels independently because of their brittleness. Other high toughness and high elastic materials are needed to improve the mechanical strength of MXenes based aerogels. For instance, Ma Y. et al. (2018) composite graphene oxide with Ti_3_C_2_T_x_ to prepare MXene/reduced graphene oxide (MX/rGO) hybrid structures. As is shown in Figure 4B, the rGO layer with a larger surface area provides a high mechanical strength skeleton for aerogels, while a better conductive Ti_3_C_2_T_x_ enhances the resistance effect of the pressure sensor. The synergistic interaction between the two materials endows the sensor with excellent sensing performance. Similarly, Liu et al. (2018) mixed Ti_3_C_2_T_x_ dispersions with poly (amic acid) (PAA) and obtained MXenes/polyimide aerogels after freeze-drying and calcination (MXenes/PI aerogel). The sensor possesses high elasticity and low density, which can sustain compression, bending, and torsion deformation. Zhuo et al. (2019) utilized cellulose nanocrystals (CNCs) as a nano-support to connect MXenes nanosheets into a lamellar carbon aerogel with not only super mechanical performances but also ultrahigh linear sensitivity (Figure 4C). Chen et al. (2019) used bacterial cellulose fiber as a nanobinder to connect MXenes (Ti_3_C_2_) nanosheets into continuous and wave-shaped lamellae to fabricated a kind of compressible and elastic carbon aerogels. Therefore, it is an effective way to prepare high-performance wearable MXenes-based piezoresistive sensors by compounding MXenes with mechanical strength materials and in *situ* growing into aerogels with high elasticity and high conductivity. Wang et al. (2019) developed a skin-inspired Ti_3_C_2_/natural microcapsule composite film with the interlocked structure that improved the mechanical deformability of the sensing layer. Mimicking the structure and function of human skin (Figure 4D), the sensor can amplify the weak pressure signal and possess excellent stability.

In addition, MXenes are directly loaded on the formed high elastic substrate, and the high conductivity of MXenes and the high mechanical properties of the elastic substrate are also used to meet the requirements of the resistance effect and geometric characteristics of the flexible piezoresistive sensor. Yue et al. (2018) prepared MXene-sponge by dip-coating process and made a piezoresistive sensor by combining it with insulated polyvinyl alcohol (PVA) nanowire. The MXene-sponge piezoresistive sensor has ultrahigh sensitivity. The GF in the pressure range of 0–5.37 and 5.37–18.56 kPa is 147 and 442, respectively. Li X.-P. et al. (2019) used the same method to load MXenes lamellae onto the skeleton of a polyurethane (PU) sponge treated with chitosan. Because chitosan is positively charged and the MXenes lamellae surface is negatively charged, MXenes lamellae can be evenly and tightly adsorbed on the sponge. Guo et al. (2019) impregnated the MXene sheet on porous fabric and constructed a sandwich structure with two layers of degradable polylactic acid (PLA) sheet to assemble a transient pressure sensor. In addition, the sensor can be completely degraded after soaking in sodium hydroxide for more than 14 days.

## Chemical Sensor

Besides excellent electronic properties, MXenes are essentially hydrophilic due to their surface functional groups, which endows MXenes great prospect in the field of the wearable sensor. MXenes can selectively adsorb biomolecules (e.g., glucose, dopamine) and gas molecules (e.g., NH_3_, NH_4_) through morphology control and surface modification, thus changing their electrical properties. Meanwhile, the major elements of MXenes (the M-layer elements) are some of the early transition metals, such as Ta, Ti, and Nb, which are relatively inert to biological organisms, which endows MXenes compounds with excellent biocompatibility. *In vivo* experiments carried out by Lin et al. (2017) showed that MXenes could be degraded and eliminated from the body of mice.

### Biosensor

**Figure 5:**
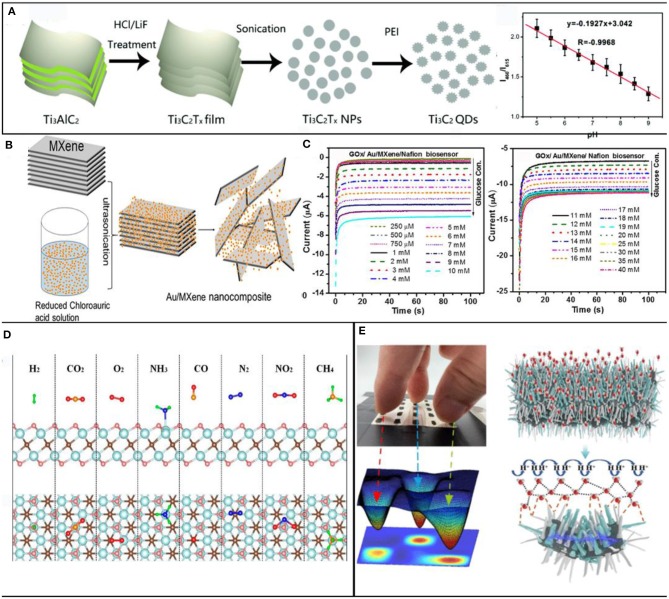
MXenes for biosensor. **(A)** Schematic illustrations for preparing the Ti_3_C_2_ QDs by using a liquid exfoliation and solvothermal treatment approach; reproduced from Chen et al. (2018) with permission from The Royal Society of Chemistry. **(B)** Schematic showing the synthesis process of Au/MXene nanocomposites; **(C)** GOx/Au/MXene/Nafion/GCE at a constant voltage of−0.402 V; reproduced from Rakhi et al. (2016). **(D)** Application of MXenes for detection of NH_3_. **(D)** is reproduced from Yu et al. (2015) with permission from American Chemical Society. **(E)** Schematic of the adsorption process of water molecules at the Ti_3_C_2_/TiO_2_ composite film. Reproduced from Li N. et al. (2019) with permission from American Chemical Society.

Recently, MXenes had been proven to be a potential intracellular pH sensor. Chen et al. (2018) fabricated a pH-sensitive Ti_3_C_2_ quantum dots (QDS), and they developed a ratiometric photoluminescence probe to monitor intracellular pH, which can be applied as a promising platform for developing wearable practical fluorescent nanosensors (Figure 5A). Besides monitoring intracellular pH, MXenes has also been designed for the detection of other small molecules, such as glucose and phenol. RAKhi reported an Au/MXenes composite biosensor platform for the detection of sensitive enzymatic glucose (Rakhi et al., 2016). The biosensor utilizes the unique electrocatalytic performance and synergistic effect between Au nanoparticles and MXenes nanosheets. Glucose oxidase (GOx) enzyme was immobilized on Nafion gold/MXenes nanocomposite and placed on the glassy carbon electrode to prepare current glucose biosensor (Figures 5B,C). The device exhibited excellent electrocatalytic activity toward a low detection limit of 5.9 μM and a wide linear range of detection of glucose from 0 to 18 mM. Li M. et al. (2019) fabricated a 3D porous MXenes-based composite for non-enzymatic glucose sensor. The 3D porous structure of Nickel-Cobalt layered double hydroxide (Nico-LDH) has a high specific surface area and many ion diffusion channels. They exported MXenes/Nico-LDH nanocomposite with a wide linearity range (0.002–4.096 mM) and a low limit of detection (0.53 μM). Novel MXenes-based nanocomposite can detect dopamine (DA), Zheng et al. (2018) synthesized a novel nanomaterial (MXenes/DNA/Pd/Pt) and applied for the development of sensitive DA sensors. Mxenes nanoparticles are used as the conductive matrix of Pd/Pt nanoparticles. DNA is adsorbed on the surface of MXenes by hydrophobic aromatic group, which induces the *in-situ* growth of PdNPs and Pd/Pt nanoparticles. The sensor exhibited excellent linearity in the DA concentration range of 0.2–1,000 μM and high selectivity against ascorbic acid, uric acid, and glucose. More interestingly, H_2_O_2_ can effectively oxidize the surface functional groups of MXenes, thus significantly increasing the oxygen density of the MXenes surface and promoting the charge transfer process. Wang et al. (2015) developed a new type of accordion-like TiO_2_-Ti_3_C_2_ nanocomposites, and they immobilized hemoglobin (Hb) on this system to fabricate a mediator-free biosensor. The TiO_2_ nanoparticles are loaded on the Ti_3_C_2_ layers substrate to form a sensing platform, which is suitable for enzyme immobilization (Liu et al., 2015).

Besides small molecules, the interaction between metal ions and MXenes produces a similar doping effect. Zhu et al. (2017) studied the electrochemical response of MXenes for the detection of the coexistence of Cd^2+^, Pb^2+^, Cu^2+^, and Hg^2+^, and proposed a new platform for sensitive detection of heavy metal ions. The detection platform with a detection limit of 0.098, 0.041, 0.032, and 0.130 mM for Cd (II), Pb (II), Cu (II), and Hg (II), respectively. In addition to detect heavy metal ions, MXenes nanosheets also possess the ability to remove heavy metals (e.g., Cu, Li, Na, K atoms) (Guo et al., 2015; Shahzad et al., 2017).

### Gas Sensor

In the field of wearable electronics, especially in the field of e-skin, in addition to the sensing demand for force and biological information, it is desirable to be able to transfer environmental factors significantly and gas sensing is a significant challenge for the next generation of wearable sensors (Ma Z. et al., 2018). The unique surface structure of MXenes is very suitable for adsorbing various gas molecules, thus affecting its overall conductivity.

The adsorption/desorption process results in the change of surface electric state of MXenes, and gas absorption can occur at the active defects on the surface of MXenes, or it can be the result of interaction with surface functional groups (Ghosh, 2014). With functional groups, gas adsorption caused by electrostatic force results in relatively small resistance changes due to the weak intermolecular force. Gas absorption may also be due to the substitution of surface functional groups by gas molecules, which leads to the carrier transfer between adsorbent and adsorbate gas, and significant changes in the resistance of materials (Geistlinger, 1993). Yu et al. (2015) have theoretically predicted the potential of MXenes-based composite in gas sensing (H_2_, O_2_, CO_2_, CH_4_, NH_3_, et al.) by first-principles simulation. They found that the Ti_2_C monolayer with oxygen terminations was more selective for NH_3_ than other gas molecules (Figure 5D). Xiao et al. (2016) considered the interaction between NH_3_ and O-terminated semiconducting MXenes (M_2_CO_2_, M=Sc, Ti, Zr, and Hf) with different charge states utilized first-principles simulations. Due to the NH_3_ can be adsorbed on M_2_CO_2_ with charge transfer, the potential of MXenes-based semiconductor as the NH_3_ sensor or capturer is revealed. Lee et al. (2017) utilized TiC_2_T_x_ integrated on flexible polyimide platforms by solution casting method. The sensor performance great in NH_3_ detection and the great potential of MXenes as a gas sensor is predicted theoretically. Lee et al. (2020) utilized a scalable wet-spinning process to prepare a Ti_3_C_2_T_x_/graphene hybrid fibers that possess excellent mechanical and high electrical conductivity. The optimized bandgap, synergistic effect, and the increased oxygen content in MXenes end atom of Ti_3_C_2_T_x_/graphene hybrid fiber significantly improve the NH_3_ sensing response. Kim et al. (2018) demonstrated Ti_3_C_2_T_x_ MXenes film as metallic channels for volatile organic compounds (VOCs) gas sensors with a high signal-to-noise ratio. Lee et al. (2019) dropped the V_2_CT_x_ solution on the flexible polyimide substrate to form a gas sensor with high sensitivity toward nonpolar gas. Yuan et al. (2018) fabricated high-performance and flexible VOC sensors. The sensor based on the 3D MXenes framework, which was prepared through electrospinning aqueous solution of the positively charged polymer. The sensor exhibited high sensitivity, good flexibility, and wide sensing range. Zhao et al. (2019) utilized polyaniline (PANI) decorated on Ti_3_C_2_T_x_ nanosheet surface via in *situ* polymerization at low temperature for a PANI/Ti_3_C_2_T_x_ composite. The synergistic properties of composites and highly active Ti_3_C_2_T_x_ endow the sensing material both high ethanol sensitivity (41.1%, 200 ppm) and rapid response/recovery time (0.4/0.5 s) at room temperature. Interestingly, MXenes not only possess excellent performance in gas sensing but also in temperature sensing. Chen et al. (2015) synthesized 2D vanadiumcarbide (V_2_C) phase by referring to the previous preparation process (Naguib et al., 2013), then graft poly(2-(dimethylamino) ethyl methacrylate) (PDMAEMA) brushes on V_2_C materials through self-initiated photographing and photopolymerization (SIPGP). Carbon dioxide and temperature can be used as stimulants to adjust the dispersion state, transmissivity and conduction activity of the system to realize the double correspondence between them.

Sensitivity to water molecules in the atmosphere is also an important factor for the MXenes-based sensor. Since the surface of MXenes is hydrophilic and the interaction between layers is relatively weak, water molecules can be inserted spontaneously under the environmental humidity, which has great potential as the humidity sensor. It has been proven that the resistivity of Ti_3_C_2_ film increases linearly by 15–80% with the increase of the relative humidity (RH) (Römer et al., 2017). Metal ion intercalation has a great influence on MXenes structure and internal surface hydrodynamics (Ghidiu et al., 2016; Osti et al., 2016). Muckley et al. (2017) demonstrated that the electrical and weight responses of K^+^ and Mg^2+^ intercalated Ti_3_C_2_ films to water vapor were between 20 and 80% RH. They further studied the gravimetric response of the intercalated MXenes to water, and found that the gravimetric response to water is 10 times faster than its electrical response. This is explained the expansion/contraction of the channel between MXenes sheets induced by water molecules results in the capture of water molecules as charge consuming dopants (Muckley et al., 2017). Yang Z. et al. (2019) alkaline-treated the Ti_3_C_2_T_x_ synthesized from Ti_3_AlC_2_ by sodium hydroxide solution. The insertion of alkali metal ions and the increase of the ratio of oxygen to fluorine on the surface can effectively improve the humidity and gas sensitivity at room temperature. An et al. (2019) utilized LbL assembly to prepare the MXene/polyelectrolyte multilayer films that possess ultrafast recovery and response times. When the humidity is changed, water molecules are inserted into the MXene/polyelectrolyte multilayers, resulting in the increase of thickness and the distance between the sheets, thus changing the tunneling resistance between MXenes layers. Li N. et al. (2019) utilized alkali oxidation method to grow in *situ* TiO_2_ nanowires on Ti_3_C_2_ to fabricate the urchin-like Ti_3_C_2_/TiO_2_ composite. The staggered dendritic nanowire structure has excellent adsorption performance at low RH, which is conducive to the formation of a continuous water layer (Figure 5E). Liu et al. (2019) developed a vacuum-assisted LbL assembly using AgNW with MXenes sheets to fabricate a highly conductive leaf-like composite on the silk substrate. The Mxenes layer protects AgNW from oxidation and endows textiles with high sensitivity to humidity, which has tremendous potential in intelligent garments and sensor applications.

## Summary and Future Prospects

MXenes materials gradually occupy a place in the field of wearable sensors because of its excellent conductivity, mechanical properties, hydrophilicity, and ease to control the morphology. In recent years, various kinds of sensors based on accordion-like MXenes materials have revealed that the conductive sensitive material structure, sensing mechanism, and sensor performance analysis have made good progress. By fully considering the advantages of MXenes materials and the target requirements of devices, a new sensing system is formed by combining MXenes materials with other suitable materials, which can maximize the synergistic effect between MXenes and other phase materials, and thus obtain a high-performance sensor with high sensitivity and wide response range.

However, in order to realize the application requirements of MXenes-based sensors in wearable devices, medical detection, and electronic skin, there are still many problems to be solved. The preparation process of MXenes usually requires the use of fluorine-containing reagents, which are toxic to the biological environment. Meanwhile, the widely applied HF etching solution is harmful to organic organisms, and the trace residue will lead to an irreversible effect on the human body. It also has high requirements for the safety and wastes liquid treatment in the mass production process. Therefore, how to realize the fluorine-free preparation is the key to make MXenes practical. For the MXenes-based sensor, it is unable to realize linear induction in a large strain range, which affects the programmed setting of the sensor in practical application. Therefore, it is necessary to further design the microstructure of MXenes and its composite materials to improve the linearity of the sensor. And for MXenes-based biosensor, although several studies have shown that MXenes currently used in biomedical applications are generally biocompatible, some of them can even be biodegraded *in vivo*. But the long-term biosafety of MXenes has not been systematically evaluated. And we still need to fully understand the surface chemistry of MXenes. Understand which functional groups exist on their surface and explore the various properties of functional groups. In addition, the etching of a elements

other than Al needs to be explored to cover all possible ternary carbides and nitrides. Meanwhile, MXenes-based transparent electronic conductors also need to explore. Therefore, it is urgent to explore ways to improve this demand of MXenes for further exploration.

## Author Contributions

MX wrote the manuscript. JL and ZM modified the manuscript. LP and YS supervised the manuscript.

## Conflict of Interest

The authors declare that the research was conducted in the absence of any commercial or financial relationships that could be construed as a potential conflict of interest.
